# Practical applications of studies on the TSH receptor and TSH receptor autoantibodies

**DOI:** 10.1007/s12020-019-02180-9

**Published:** 2020-05-29

**Authors:** J. Furmaniak, J. Sanders, P. Sanders, J. Miller-Gallacher, M. M. Ryder, B. Rees Smith

**Affiliations:** 1 FIRS Laboratories, RSR Ltd, Cardiff, UK; 2 Mayo Clinic, Rochester, NY USA

**Keywords:** Thyroid, Graves’ disease, TSH receptor, Autoantibodies, Graves’ orbithopathy, Thyroid cancer

## Abstract

Studies on the TSH receptor (TSHR) have numerous practical applications in vitro and in vivo. For example human monoclonal autoantibodies (MAbs) to the TSHR are useful reagents for in vitro diagnostics. Measurement of TSHR autoantibodies (TRAbs) is helpful in diagnosis and management of autoimmune thyroid disease. Currently available highly sensitive and specific assays to measure TRAbs use the human TSHR MAb M22 instead of the TSH. Furthermore, preparations of the human TSHR MAb M22 are useful as the World Health Organisation International Standard for thyroid stimulating antibody and for calibration of the assays for measuring TRAbs. Preparations of thermostabilised TSHR extracellular domain have recently become available and this is likely to have an impact on improvements in specificity testing for TRAb assays. In addition the stable TSHR preparations have practical application for specific immunoadsorption of patient serum TRAbs. Human TSHR MAbs also have promising prospects as new therapeutics. Autoantibodies with TSHR antagonistic activities are “natural” inhibitors of TSHR stimulation and are expected to be helpful in controlling TSHR activity in patients with Graves’ disease, Graves’ ophthalmopathy and thyroid cancer.

## Introduction

The presence in patient sera of the long-acting thyroid stimulator (LATS), distinct from TSH, was first described in 1956 by Adams and Purves [1]. However, the mechanism of thyroid stimulation by LATS that was found to be associated with the IgG fraction of serum proteins was not fully understood. Almost two decades later in 1974, pivotal studies by Smith and Hall showed that these autoantibodies in sera of patients with Graves’ disease target the TSH receptor (TSHR) and stimulation of the TSHR by autoantibodies is responsible for thyroid overactivity in Graves’ disease [2]. This key observation resulted in development of the first in vitro receptor binding assay to measure TSHR autoantibodies (TRAb) to help in the diagnosis and management of autoimmune thyroid disease (AITD). The next important milestone in studies on the TSHR was cloning of the TSHR gene in 1989–1990 in four independent laboratories [3–7]. More than a decade later (in 2003) the human thyroid stimulating monoclonal autoantibody (termed M22) was isolated from the peripheral blood lymphocytes of a patient with Graves’ disease [8]. Further human monoclonal autoantibodies (hMAbs) to the TSHR were isolated soon thereafter; two hMAbs with TSHR blocking activity (5C9 and K1-70) and another stimulating hMAb (K1-18) [9]. Advances in recombinant TSHR gene expression combined with the availability of hMAbs culminated in crystallising the complexes of the TSHR leucine rich repeat domain (LRD) with M22 Fab and with K1-70 Fab [10, 11]. These solved structures provided for the first time a unique insight into the molecular structure of the TSHR LRD and into the molecular interactions of the TSHR LRD with the stimulating hMAb M22 and with the blocking hMAb K1-70 [10, 11]. Separate developments resulted in producing TSH responsive functional thyroid follicles in vitro opening new prospects for regenerative therapies for patients requiring long term thyroid hormone replacements [12, 13]. Furthermore, a long awaited mouse model of Graves’ ophthalmopathy (GO) was finally described [14]. Very recently thermo-stable preparations of the TSHR LRD were obtained and for the first time the crystal structure of a ligand-free glycoprotein hormone receptor domain was solved (2.83 Å resolution) [15]. All of these considerable scientific achievements are leading to improvements in diagnosis, monitoring and management of patients with AITD.

## In vitro practical applications of TSHR antibodies

Once it had been demonstrated that stimulation of the TSHR by autoantibodies in patients’ sera has a central role in the pathogenesis of Graves’ disease measurement of TRAb for diagnosis and monitoring of patients has become increasingly important. The methods available to measure TRAb have evolved over the years. First generation liquid phase assays were based on inhibition of ^125^I-labelled TSH binding to TSHR preparations by serum TRAb followed by precipitation using polyethylene glycol [16]. These assays were a milestone in diagnosis of Graves’ disease providing a convenient alternative to time consuming and complex bioassays. Second generation assays used solid phase technology where TRAb in a test sample bind to TSHR preparations immobilised on ELISA plate wells or plastic tubes prior to adding TSH labelled with biotin [17] or ^125^I. The solid phase assay design including important wash steps between additions of key reagents, enabled the use of non-isotopic labels and automation and resulted in increased assay sensitivity and specificity compared to first generation assays. In third generation assays M22-biotin is used in place of TSH-biotin leading to further improvements in sensitivity and specificity [18]. M22 has a particular advantage over TSH in the TRAb inhibition assays as M22 is not easily dissociated once bound to the TSHR. This is especially useful in automated systems which require for the ligand to remain tightly bound during stringent washing procedures. Third generation TRAb assays are now used widely worldwide on various platforms. They show excellent sensitivity and specificity even with assay times of <30 min [18, 19].

The concentration of TRAb in the assays is expressed in international units per litre relative to the World Health Organisation (WHO) reference preparations supplied by the National Institute for Biological Standards and Control (www.nibsc.org). The WHO International Standard for thyroid stimulating antibody is a useful reagent for calibration of bioassays and receptor assays for measuring of TRAbs. The 1st Standard (coded 90/672) was obtained from a patient donor and eventually ran out. Availability of M22 made it possible to prepare a 2nd International Standard (coded 08/204). Each ampoule contains 1 μg of freeze-dried M22 IgG in normal human serum and the Standard 08/204 is currently widely available. Furthermore, essentially unlimited amounts of M22 IgG for production of the 08/204 Standard are assured and consequently the supply should never run out.

## In vitro practical applications of TSHR research

One of the features of the TSHR molecule is that it is inherently unstable and difficult to purify to homogeneity. These difficulties have been overcome in part be stabilising the TSHR LRD with M22 Fab or K1-70 Fab and resulted in solving the molecular structures of the respective complexes [10, 11]. However until very recently purification of the TSHR LRD without an autoantibody bound had not been successful due to poor stability of the un-complexed protein. A strategy of thermostabilisation by systematic site-directed mutagenesis has proven very useful in obtaining stable TSHR LRD preparations. Amino acids in the human TSHR sequence from 22 to 260 (TSHR260) were systematically mutated and the thermostability of the protein tested under the stressed conditions of heating at 42 °C. The most thermostable mutations were then combined in a series of experiments until the most thermostable mutant containing six different mutations was obtained. This mutant was termed TSHR260-JMG55 and it is about 900 times more stable than the wild type TSHR260 [15]. The responses of the full length TSHR with the respective part of the sequence replaced by TSHR260-JMG55 to stimulation by TSH or M22 were similar to that of the wild type. Furthermore, the full length TSHR containing mutated TSHR260-JMG55 domain showed similar ability to wild-type TSHR to bind TSHR autoantibodies present in different Graves’ patient sera [15].

The TSHR260-JMG55 was purified to homogeneity, deglycosylated and crystallised. The crystals diffracted to 2.83 Å resolution allowing for the structure of a ligand free TSHR260 to be solved by molecular replacement [15]. The solved TSHR260-JMG55 structure showed remarkable similarity to the crystal structures of the TSHR260 from TSHR260–M22 and TSHR260–K1-70 complexes. This indicates that neither the mutations nor the binding of the autoantibodies change the rigid LRD structure of the TSHR.

The availability of stable, autoantibody binding TSHR preparations have several potential practical applications. Stable concentrated TSHR260-JMG55 can serve as a useful reagent for specificity testing in TRAb receptor assays and bioassays. Appropriate adsorption protocols are now available in which samples giving equivocal results in the TRAb assays would be incubated with the TSHR260-JMG55 preparations. If the true TRAb are present in the test sample these are adsorbed by TSHR260-JMG55 and the results verified as positive. In addition, TSHR260-JMG55 may be useful in developing new assays for measurements of TRAbs, which may be based on different principles than the binding inhibition assays currently in use.

## In vivo practical applications of TSHR research

An important potential practical application of stable TSHR preparations in vivo is for the specific immunoadsorption of patient serum TRAb. In critical clinical situations requiring urgent depletion of TRAb from patient’s circulation passaging the plasma through a matrix with TSHR260-JMG55 bound to it would remove the patient’s TRAb thus providing a quick and effective therapeutic effect. Finally, stable pure TSHR260-JMG55 should be a valuable reagent for studies on immune responses to the TSHR.

The human Mab K1-70 with TSHR blocking activity is a powerful inhibitor of TSHR stimulation by both TSH and by stimulating antibodies. As such K1-70 is a “natural” inhibitor of TSHR stimulation [20]. There are several practical in vivo applications of targeting the TSHR with K1-70 including controlling thyroid overactivity in Graves’ disease and GO. In addition, blocking TSHR activity with K1-70 may benefit patients with thyroid cancer and Graves’ disease [21–23]. Furthermore, the high binding affinity and specificity of K1-70 for the TSHR may be useful for imaging of tissues expressing the receptor and targeted drug delivery.

A different novel strategy to control thyroid cancer includes targeting the TSHR with nano-liposomes coated with fragments of TSH and loaded with chemotherapeutic agents [24]. Another proposed approach is based on using TSHR binding carbon-walled nanotubes, which have the ability to convert external light energy to heat energy resulting in killing the cancer cells locally [25]. Also, small molecule antagonist to the TSHR would offer yet another approach to inhibit thyroid cancer progression by suppressing TSHR signalling [26]. Currently there is an ongoing phase 1 safety, tolerability, pharmacokinetic and pharmacodynamics first in human clinical trial in patients with Graves’ disease with K1-70 administered in a single ascending dose (https://clinicaltrials.gov/ct2/show/NCT02904330). In addition, a request for expanded access to the drug was made for a female patient with Graves’ disease, severe GO and locally advanced and distant metastatic well-differentiated follicular thyroid cancer [27, 28]. Before administration of K1-70, the patient’s GO clinical activity score (CAS) was 6/7, she had diplopia, exophthalmometry of 21 mm bilaterally with chemosis, injection, lid swelling, pain with eye movement and lid erythema. She was initiated on 3-weekly administrations of K1-70 in doses designed to neutralise her high levels of thyroid stimulating autoantibody. At the time of first dosing TRAbs were 130IU/L with thyroid stimulating activity >2000% stimulation. The patient was also on lenvatinib for her thyroid cancer. She reported improvement in her eye signs soon after initiation of K1-70 therapy; CAS improved to 0–1/7 and exophthalmometry to 19 mm bilaterally allowing for surgical correction of the diplopia. The patient was able to resume driving and GO remained inactive while on K1-70 therapy. Furthermore, attenuation of tumour progression was observed on K1-70 alone while lenvatinib was withdrawn due to side effects. There have been no observed adverse effects of K1-70 after more than 24 months of treatment at up to 120 mg per dose.

These early observations indicate that blocking TSHR activity in vivo with K1-70 has promising applications in controlling stimulation of the receptor in Graves’ disease, GO and in thyroid cancer.
